# Bridging the Language Gap in Healthcare: Implementing a Qualified Medical Interpreter Program for Lesser-Spoken Languages

**DOI:** 10.3390/ijerph21101377

**Published:** 2024-10-18

**Authors:** Michelle Mavreles Ogrodnick, Mary Helen O’Connor, Coco Lukas, Iris Feinberg

**Affiliations:** 1 Department of Learning Sciences Georgia State University, Atlanta, GA 30302, USA; ifeinberg2@gsu.edu; 2 Independent Consultant, Orlando, FL 32804, USA; maryhelenoconnor@gmail.com; 3 Health Literacy Consultant, Roswell, GA 30075, USA

**Keywords:** qualified medical interpreter, limited English proficiency, social determinants of health, health equity, refugee and immigrant health

## Abstract

Linguistic inequity drives systemic disparities in healthcare for non-native English speakers. This study evaluates a project to train and provide qualified medical interpreters (QMI) to assist volunteer and safety-net clinics and community-based organizations in supporting healthcare for immigrants and refugees. We provided scholarships to bilingual community members to take a medical interpreter training course and developed a workforce for those who passed the training course. We focused on lesser-spoken foreign languages such as Arabic, Amharic, Pashto, Dari, and Burmese. Those who passed the course participated in a semi-structured interview to learn about their experiences in the training program, as well as barriers and facilitators to becoming a QMI. To date, 23 people have passed the training and are part of the QMI workforce program that has provided 94 h of interpreter services over four months, serving 66 individual patients. The evaluation showed that community members have interest in becoming QMIs and many have the required language proficiency to enroll and pass training. Finding full-time employment for less spoken languages has proven to be challenging.

## 1. Introduction

Social determinants of health (SDOH) are primary drivers of structural health disparities especially among marginalized and minority populations including immigrants and refugees [1]. Healthy People 2030 has centered their approach on achieving health equity on social determinants including safe housing, access to transportation and nutritious food, education, and employment [2]. Language is a key social determinant that intersects and influences all other SDOH [3]. Virtually every aspect of a person’s psychological, physical, and behavioral life is affected by the ability to communicate in the predominant language in the country of residence [4]. Thus, for the 22% of the U.S. adult population who reported speaking a language other than English at home [5], linguistic inequity drives systemic disparities, especially in healthcare [6]. Whether communicating with a healthcare provider, understanding instructions for a medication, applying for financial assistance or insurance, or even making an appointment, language and literacy barriers exacerbate poor health outcomes for non-native English speakers (NNES) who access the U.S. healthcare system. NNES have lower rates of insurance, suffer higher rates of chronic disease, and overall have poorer health outcomes than native English speakers [7,8].

Understanding how language proficiency affects health and well-being is complex. Systematic reviews of the literature reveal many gaps in what has been researched and learned about NNES populations. The collection of data from NNES patients in the U.S. has not been consistent or standardized [9]; a recent change from the Office of Management and Budget expanding race and ethnicity data collection standards in 2024 is the first meaningful data collection change since 1997. Populations who are on the move, have limited literacy, or are mistrustful of institutions and government, also present challenges to researchers [10,11]. It is challenging, expensive, and time-consuming to develop research instruments in multiple languages and cultures [12]. When researching healthcare providers, stigmatized cultural beliefs are also not easily elicited [13]. While there is compelling research establishing the significance of language proficiency on health outcomes, very few studies consider the impact of the use of trained, qualified medical interpreters (QMI) on quality of care [6]. What data have been collected indicate that patients who are provided a QMI, or who have a bilingual provider, have fewer emergency department visits, lower hospital readmission rates, and higher satisfaction compared with those who have no language support [6,14]. In a systematic review of studies investigating language support in clinical settings, the findings include serious adverse impacts associated with the use of untrained informal interpreters (e.g., family, friends, volunteers, Google translate) including violations in confidentiality, miscommunication of diagnoses and care plans, and incomplete instructions for the administration of medication including expected side effects, as well as serious consequences for patients with mental and behavioral health concerns [6,15,16,17]. The quality of healthcare for NNES is compromised when trained interpretation services and language support are not utilized. Patients who are not provided adequate language support have an incomplete and inadequate understanding of their diagnosis and care plan; untrained or informal interpreters “misinterpret or omit up to half of all physicians’ questions” and are more likely to make mistakes with serious clinical consequences [6] (p.273). Patients with mental and behavioral health needs are disproportionately impacted by a lack of language concordance [18,19].

Studies indicate an overall improvement in medical care and health outcomes when QMIs are utilized [14,20]. Despite the prevailing narrative in the U.S. that medical interpretation services are exorbitantly expensive, these studies also suggest that the costs associated with using language support services are generally low especially considering poor clinical outcomes resulting from mistakes made by not using interpretation support [6,14,21]. Considering the legal obligation of healthcare providers who receive federal funds to provide interpretation support to any patient requesting it (Title IX of the Civil Rights Act of 1964) as well as recommendations from the Department of Health and Human Services Culturally and Linguistically Appropriate Services Standards (CLAS), providing qualified language support is still an unfunded and unenforced mandate with considerable repercussions for NNES patients [22,23]. Research suggests that NNES patients who are not provided with a trained interpreter use fewer outpatient services, have poorer outcomes than English-proficient patients with diabetes, ask fewer questions of their providers, and overall experience more pain, have worse psychological well-being, and have lower levels of patient satisfaction [4,24,25,26,27,28]. Close to 2 million people annually receive care in smaller community-based sliding-scale or volunteer free clinics or local boards of health which are under-represented in research studies; 84% of this population are immigrants or refugees [29].

There remains a significant need to explore the impact of language and literacy to improve quality of care and reduce health disparities for NNES. This study seeks to contribute to what is known about the efficacy and impact of providing QMIs for lesser-spoken language NNES in small community-based settings where the resources to provide quality interpretations are limited. In this study, we evaluate a project to train and provide QMIs to assist local clinics and community-based organizations (CBOs) in supporting healthcare for immigrants and refugees.

## 2. Setting

The study setting is DeKalb and Fulton Counties which are the two largest counties in Georgia and have a combined documented and undocumented foreign-born population of 330,000 [30,31,32]; 37.4% report being a limited-English-proficient household. More than 1/3 (38.5%) of NNES in both counties are Spanish speaking [30]; however, DeKalb County is also home to the city of Clarkston, a multi-ethnic community where more than 60,000 refugees have settled since 2004 [33]. Currently, half of Clarkston’s 14,000 residents are foreign-born refugees who speak more than 60 languages including Amharic, Arabic, Burmese, Dari, English, French, Karen, Pashto, and Swahili. Due to the numerous languages spoken in the area, there is a need to focus not only on the larger language groups but on some of the less used languages.

## 3. Materials and Methods

We provided scholarships to bilingual community members by sharing a flyer with CBOs beginning in October of 2023. Scholarships were provided on a first come first served basis regardless of the known language to encourage inclusivity. Interested community members responded by completing a Google form. Requirements to become a QMI included proving bilingual fluency by providing both a high school degree or college diploma where all coursework took place in English and the same in a non-English language. If an applicant did not have a degree/diploma in one of the two languages, they were eligible for a scholarship to take a listening and speaking fluency test. The fluency test took place over the phone and was approximately 30 min long; it was a prerecorded test for most languages (due to limited usage, Swahili and Kinyarwanda were performed with a live proctor). Two evaluators reviewed the applicant’s fluency test and provided a score indicating the applicant’s level of fluency. Applicants had to score an eight or higher on a scale of 1 to 12 to be considered bilingually fluent. The scholarship funds were paid directly to the company offering fluency testing.

Applicants with proven bilingual fluency were eligible to receive a scholarship to complete a 40 h medical interpretation course with a final exam. The scholarship funds were paid directly to the company offering the medical interpreter training course. Two courses were offered: one was a 40 h virtual synchronous course over 5 weeks offered by the Language Interpreter Services department of a local hospital (Bridge the Gap, BTG) [34] and the other was a 40 h virtual asynchronous course offered by a professional language services company which could be completed at candidate’s own pace over 4 months (Breaking Boundaries in Healthcare, BBIH) [35]. BTG required approval of fluency documentation by the training provider or a fluency test score of 10 or higher. Candidates who received a fluency test score of 8 or higher were able to take BBIH. Both courses focus on interpreting skills, ethics, medical terminology, working in a healthcare setting, and the role of culture in interpreting. Each applicant needed a laptop with a video camera and headset to complete the medical interpreter training course. Participants who did not have access to the necessary equipment were able to borrow it from the research team.

Participants who completed the course were invited, via email, to participate in a semi-structured interview to learn about their experience in the training program and gain a better understanding of the barriers and facilitators in becoming a QMI. See Appendix A for the semi-structured interview guide. Interview participants received an electronic copy of the informed consent to review before the interview. The interviews took place virtually using TEAMs and lasted approximately 20 min; the semi-structured interviews were recorded and transcribed. Participants received a USD 25 e-payment through Zelle or CashApp for their time. The Georgia State University institutional review board approved this study.

We then created an interpreter workforce program matching QMIs with clinics utilizing a WhatsApp group chat. Participating clinics could request a specific language QMI for either a walk-in appointment or a scheduled appointment; if a QMI was available, they responded, and details were arranged between the clinic and QMI for interpretation services. QMIs received USD 25 per hour compensation for their time with a minimum of one hour payment.

## 4. Results

### 4.1. Scholarships and Training Process

Between October 2023 and August 2024, 144 applicants completed a QMI interest form; 61.8% (*n* = 89) provided fluency documents; of those, 24.7% (*n* = 22) provided documentation in both languages and 75.3% (*n* = 67) needed a fluency test in either English or a native language. Of those who needed a fluency test, 76.1% (*n* = 51) took a test and 78.4% (*n* = 40) passed with a score of 8 or higher.

BTG was offered over a five-week period in both February and June of 2024; 21 participants took the course with a passing rate of 95%. BBIH was offered continuously between October 2023 and November 2024; 17 applicants took the course. Of the 17 BBIH participants, two people have passed the course, six have failed or did not complete the course, and nine are still taking the course. The languages and numbers of participants for both courses include Amharic (2), Arabic (4), Bengali (4), Burmese (4), Dari (6), French (5), Haitian Creole (1), Kinyarwanda (1), Pashto (1), Russian (1), Spanish (7), Swahili (1), and Ukrainian (1).

We interviewed 15 QMIs: 13 took the synchronous BTG course and 2 took the asynchronous BBIH course. Interview participants were 19 to 53 years old with time in the U.S. ranging from 8 months to 35 years. Interview transcripts were analyzed using thematic content analysis. A researcher and the principal investigator of this study collaborated to develop an initial coding manual. Two coders independently coded the same two transcripts and discussed inconsistencies to refine the coding manual. The process continued until strong inter-rater reliability was achieved (kappa = 8; percentage agreement = 88%). The remaining transcripts were split among the coders, who discussed questions to maintain coding consistency. See Table 1 for a summary of facilitators and barriers to becoming a QMI.

There were five key takeaways from the semi-structured interviews. First, the majority (*n* = 14) of participants did not have trouble providing documentation to show proof of fluency in at least one language. Participants stated something similar to this participant who said

**Participant 10:** *“Mmm, no. Uh, I didn’t have a problem in that regards. Uh. They asked the, like a high school degree or university, college degree. Uh, and I had uh, I have one from the U.S. and from Afghanistan. Uh, I shared one of them and it was sufficient for them.”*

Second, participants reported personal challenges to taking the course. Participants discussed how childcare, school, work, and religious practices made completing the course a challenge. For example, participants stated

**Participant 2:** *“…for me I would prefer it to be in the middle of the week because at the weekend my toddler was with me at the house.”*

**Participant 10:** *“It was during the weekend so, my, I have a son, 2-year-old son. He was at home. He was not in daycare. During the weekdays he is in daycare.”*

**Participant 10:** *“Umm. Just one thing at, towards the end of the course, me as a Muslim, the holy month of Ramadan started, so it was kind of difficult for me to uh, like if they focus for the whole 5 h while fasting… those holidays, and like Ramadan, takes your attention away, a little bit.”*

**Participant 11:** *“I think just me trying to juggle everything because I did, I was a full-time student and I had a job and then I was trying to, you know, do that to take the test on top of it.”*

Third, all the participants said they would recommend the class to others who want to be a medical interpreter. Participants discussed how the course taught them interpreter techniques to use and gave them more confidence. For example, participants stated

**Participant 11:** *“…now I’m more confident and my applying for interpreting job because I know the standard and what I should and shouldn’t do. Yeah most of the time like I work with like refugees, so they don’t get like they don’t usually have someone who is like always helping them if their children are like at work or if they’re too little. So, they like, feel really appreciative and they will like, invite me to the house…. Just having like boundaries regarding that because that could like lead to other things.”*

**Participant 2:** *“Yeah, sure. I would say that this course will prepare you to be professional interpreter, because with “Instructor” we saw many videos about people who were trying to be like interpreter because they know the other language and English. But that was so unprofessional because sometimes you feel like you are doing the good thing, but it is not.”*

Fourth, all participants said the scholarship influenced their desire and ability and helped reduce the financial burden to take the course. For example, participants said

**Participant 8:** *“The scholarship provided me with the opportunity to pursue the course at the optimal time and with full dedication to learning and absorbing the information. It greatly influenced my ability to undertake the training course and reinforced my desire to participate.”*

**Participant 2:** *“… the scholarship opportunity has definitely influenced my decision and motivation to pursue the training course. Knowing that I have financial support for the course removes a significant barrier and allows me to fully focus on learning and preparing for a career as a medical interpreter.”*

Lastly, most participants felt prepared to be an interpreter. However, some participants said they felt somewhat or not prepared and felt like they needed more interpreting practice.

**Participant 12:** *“No. I there’s so much Spanish medical terms in Spanish that I don’t know… it’s insane the terminology. I have to memorize a lot of stuff. I don’t feel prepared.”*

**Participant 17:** *“I feel 50% prepared. I’m still looking for the practicum; being able to practice what I’ve learned … Um so I’m hoping that will give me some comfort level where I can practice the skill…”*

### 4.2. Creating a QMI Workforce

The interpreter WhatsApp workforce group was implemented in mid-May 2024. All QMIs who passed the courses signed up. There are eight clinics/non-profit groups participating, representing family practice, diabetes, maternal services, and mental health. In the first four months of operation, 114 requests for interpreters have been made with successful interpreter availability at 52% (*n* = 59). This represents interpretation for 67 patients at a range of 1–4.5 h per patient. A total of 94 interpreter hours were utilized. Interpretation services were provided in Amharic, Arabic, Burmese, Dari, Pashto, Spanish, and Swahili.

## 5. Discussion

This study evaluated the processes for becoming a QMI, as well as the supply and demand for QMIs by local clinics and community-based organizations in DeKalb and Fulton Counties. The population of NNES in the U.S. is continuing to increase and, with it, the need for appropriate language services [36]. Data show that by 2050 the U.S. demographics will be more diverse with an increase in African Americans, Hispanics, Asian Americans, and immigrant populations and a reduction in non-Hispanic whites [37]. The population is expected to grow more from international migration instead of a natural increase due to a decline in birth rates and the age of the population [38]. It is estimated that the foreign-born population will reach a historic high of 60 million people by 2060, which will be approximately 17% of the population [38].

Language barriers between patients and providers can lead to miscommunication, reduced satisfaction, decreased quality of care, and decreased patient safety [4]. Research shows that patients who receive language concordant care have greater communication, can align on treatment plans, have more satisfaction, and have fewer questions about care [20]. Additionally, patients have a clearer understanding of their diagnoses and are more likely to speak up about health problems [20]. When a patient feels like they can share concerns, it creates more productive dialogue between the patient and provider by establishing person-centered care, which ensures patients’ preferences and needs are incorporated into the care plan [39]. When language concordance occurs, it can help with the development of a trusting relationship and mutual decision making [40,41].

Ad hoc or informal interpreters, including family and friends, are frequently used to provide interpretation services during a healthcare visit [42,43] but do not have medical training and are prone to making errors [44]. Asking a family member or friend to interpret not only increases the risk of significant medical errors but can also put the ad hoc interpreter in a difficult position by asking them to deliver information they may not understand or perhaps share a difficult diagnosis [45]. Data show that ad hoc interpreters cause twice as many errors compared with qualified medical interpreters [45]. Interpretation errors can lead to serious health consequences; one study completed in an Emergency Department shows that the proportion of errors was significantly lower for professional interpreters compared with ad hoc interpreters [46]. QMIs are not only bilingual; they also receive training in medical terminology, ethics, and appropriate interpreter communication techniques [34,35]. Professional QMIs provide the highest quality of care for patients ensuring higher levels of patient safety and health equity [47].

While larger hospitals and federally funded clinics have access to professional language interpretation services, many smaller community clinics and CBOs do not. Our project focusses on the smaller clinics and CBOs that provide free or sliding-scale care and cannot afford to pay to use language line services at USD 3.95 per minute for audio calls or USD 4.95 per minute for video calls [48]. Safety-net clinics and free clinics typically operate using volunteer providers and staff with limited access to trained medical interpreters [49]. These clinics help provide crucial care to approximately two million people annually who do not have insurance, face numerous barriers to care (e.g., financial, transportation barriers), and who may distrust traditional healthcare settings [50]. One way to address this disparity is to provide QMI-training opportunities to NNES community members. Our results show that community members who speak a variety of lesser-spoken languages have an interest in becoming QMIs and many have the required language proficiency to enroll and pass training.

While the job rate growth for interpreters and translators is projected to grow 4% over the next ten years (2022–2032) [51], there are limited job opportunities for QMIs in lesser-spoken languages. In the hospital setting, there is a preference to hire certified medical interpreters. In addition to the requirements needed to become a QMI (prove fluency in at least two languages and complete a 40 h medical interpreter training course), a certified medical interpreter must pass a national exam that guarantees that the interpreters have been tested in medical interpretation ethics, procedures, and interpreting in both languages [52]. Currently, the exam is only offered in six languages: Spanish, Russian, Mandarin, Cantonese, Korean, and Vietnamese [52]. The national certification process is still relatively new when you compare it with other professions [53]. Steps are being taken to add languages to the national certification process, but it takes time and is not moving fast enough to meet the needs of interpreters or patients [52]. There is a certification credential for languages of less diffusion that tests individuals in English only but does not carry the same language distinction as a certified interpreter [54]. One challenge to certify in lesser-spoken languages is the difficulty in finding qualified individuals who can administer the national exam. For now, only six languages are eligible for national certification.

The Centers for Medicare and Medicaid Services recommended that organizations such as hospitals, health systems, and clinics use QMIs when developing a language access plan for patients [55]. Organizations may use multiple methods including full time in-person interpretation staff, contracted in-person interpreters, remote telephone interpreters, and remote video interpreters to provide language services to patients. There are pros and cons to all these methods and the organization must decide which to use based on the populations they serve [55]. If a language makes up a small percentage of the organization’s population, it is unlikely they will hire a full-time QMI just for that language. The workforce program we developed is a way to meet the needs of the lesser-spoken languages using trained QMIs. Formalizing projects that pay QMIs of lesser-spoken languages and connecting them to clinics who serve patients who speak these languages is needed to provide high quality care and remove health disparities.

## 6. Conclusions

To reduce health disparities and improve health outcomes, NNES need access to language concordant healthcare. Providing medical interpreter training scholarships to bilingual community members is one way to address the lack of available lesser-spoken language QMIs. While it is important to continue to increase the number of interpreters of lesser-spoken languages, there also needs to be a plan in place to make these services available and employ interpreters in the necessary settings. Future work should aim to secure long-term funding for workforce programs that connect QMIs to smaller clinics that provide free or sliding-scale care.

There are limitations to this evaluation. First, we focused on local clinics and community-based organizations in DeKalb and Fulton Counties. The lesser-spoken language groups in this area may not be generalizable to other communities throughout the U.S. Second, the sample size for the semi-structured interviews was relatively small. While we did invite participants who passed and failed the course to complete an interview, only participants who successfully passed the course agreed. This may have led to us missing some key barriers to the process from the participants who did not pass the course. There may have been participant response bias as participants may have been inclined to answer some questions more positively. To deter response bias, researchers made it clear that participants could skip questions they did not feel comfortable answering and that all feedback was welcome. Finally, the interpreter workforce program is in its early stages; our understanding of the demand for and availability of lesser-spoken language QMIs will continue to be assessed over another 12 months to determine the viability of the program. Further funding for the workforce program and QMI scholarships will be needed to continue this work.

## Figures and Tables

**Table 1:** Facilitators and barriers to becoming a QMI.

Facilitators	Barriers
Fluency documentation was easily accessible	Childcare
Scholarship reduced financial burden	Attending school for other learning purposes
	Work
	Religious practices

## Data Availability

The data presented in this study will be made available by the authors on request.

## Appendix A

Semi-Structured Interview Questions

1. How old are you?
2. How long have you lived in the US?
3. How did you hear about the scholarship opportunity to take the medical interpreter course?
4. Did you have any challenges providing the documents that showed you are fluent in (Language)? What were those challenges?
5. (If the person took a fluency test): You took a fluency test to show you are fluent in (Language).
  - What did you find hard/easy about taking that test?
  - Did you do anything to prepare for the test? What did you do?
  - Is there anything you wish you did to prepare for the test?
6. What did you like about the medical interpreter training course?
7. What did you not like about the medical interpreter training course?
8. Was there anything that made taking the course a challenge?
  - Time/dates of class
  - Technology issues
  - Format of the class
  - Childcare needs
9. Would you have liked to have some in-person sessions? (Why/Why not)
10. How much time outside of class did you spend preparing for class each week?
11. Would you recommend the class to others who want to be a medical interpreter?
  - Why or why not?
12. Would you have taken the medical interpreter training course if you had to pay for it yourself?
  - How much did getting the scholarship influence your desire and ability to take the training course?
13. Do you feel prepared to be a medical interpreter?
14. Are you looking for job opportunities? If so, where are you looking?
